# Resveratrol and Pterostilbene, Two Analogue Phenolic Compounds, Affect Aquaglyceroporin Expression in a Different Manner in Adipose Tissue

**DOI:** 10.3390/ijms19092654

**Published:** 2018-09-07

**Authors:** Saioa Gómez-Zorita, Jenifer Trepiana, Alfredo Fernández-Quintela, Marcela González, María P. Portillo

**Affiliations:** 1 Nutrition and Obesity Group, Department of Nutrition and Food Science, University of the Basque Country (UPV/EHU) and Lucio Lascaray Research Institute, 48940 Vitoria, Spain; saioa.gomez@ehu.eus (S.G.-Z.); jenifer.trepiana@ehu.eus (J.T.); mariapuy.portillo@ehu.eus (M.P.P.); 2 Biomedical Research Networking Centres, Physiopathology of Obesity and Nutrition (CIBERobn), Institute of Health Carlos III, 28029 Madrid, Spain; 3 Nutrition and Food Science Department, Faculty of Biochemistry and Biological Sciences, National University of Litoral and National Scientific and Technical Research Council (CONICET), 3000 Santa Fe, Argentina; maidagon@fbcb.unl.edu.ar

**Keywords:** aquaglyceroporins, white adipose tissue, brown adipose tissue, resveratrol, pterostilbene

## Abstract

Aquaglyceroporins (AQPs) are transmembrane channels that mediate glycerol release and glycerol uptake. They are involved in fat metabolism, with implications in obesity. The aim was to determine whether the administration of resveratrol and pterostilbene during the six weeks of the experimental period would modify AQPs expression in white and brown adipose tissues from Wistar rats fed an obesogenic diet, and to establish a potential relationship with the delipidating properties of these compounds. Consequently, thirty-six rats were divided into four groups: (a) group fed a standard diet; and three more groups fed a high-fat high-sucrose diet: (b) high-fat high-sucrose group: (c) pterostilbene-treated group (30 mg/kg/d): (d) resveratrol-treated group (30 mg/kg/d). Epididymal, subcutaneous white adipose tissues and interscapular brown adipose tissue were dissected. AQPs gene expression (RT-PCR) and protein expression (western-blot) were measured. In white adipose tissue, pterostilbene reduced subcutaneous adipose tissue weight and prevented the decrease in AQP9 induced by obesogenic feeding, and thus glycerol uptake for triglyceride accumulation. Resveratrol reduced epididymal adipose tissue weight and avoided the decrease in AQPs related to glycerol release induced by high-fat high-sucrose feeding, suggesting the involvement of lipolysis in its body-fat lowering effect. Regarding brown adipose tissue, AQP7 seemed not to be involved in the previously reported thermogenic activity of both phenolic compounds.

## 1. Introduction

Aquaglyceroporins (AQPs) are transmembrane channels that are permeable both to water and to small solutes, such as glycerol, urea, or nitric oxide. Due to their ability to transport glycerol through the plasma membrane, regulating cellular glycerol content, they mediate glycerol release from adipose tissue and glycerol uptake in the liver and heart. Recent studies have evidenced that AQPs are involved in fat metabolism, with implications in obesity and related co-morbidities, such as non-alcoholic fatty liver disease (NAFLD) [1,2,3,4,5].

Glycerol is the precursor for de novo synthesis of triglycerides and is also a crucial intermediate in lipid and carbohydrate metabolism [6]. Intracellular glycerol activates the enzyme glycerol kinase (GK), promoting the synthesis of glycerol-3-phosphate (G3P), which is the substrate used for triglyceride synthesis. In white adipose tissue, free glycerol is not transformed into G3P in significant proportions, due to low GK activity [7,8]. Nevertheless, in obese subjects, and under high-fat feeding conditions, the activity of this enzyme increases [7,9]. Aquaglyceroporin 7 (AQP7) significantly facilitates the rapid release of glycerol outside the adipocyte [10,11], thus successfully preventing significant intracellular recycling of glycerol to G3P and thus limiting triglyceride formation. AQP7 also plays an important role under negative energy conditions, such as fasting or energy restriction, as well as in exercise, when stimulation of lipolysis results in sharply increased free fatty acid release and glycerol from adipose tissue, which can be used by other tissues as an energy source [12].

Although AQP7 represents the main pathway in facilitating release of glycerol in adipose tissue, aquaglyceroporin 3 (AQP3) also contributes to this release, but to a lesser extent. Moreover, adipocytes also express aquaglyceroporin 9 (AQP9), which probably mediates the influx of glycerol into adipocytes [2]. Rodriguez et al. demonstrated that, under basal conditions, AQP3 is located in the plasma membrane and cytosolic fraction of adipocytes, AQP7 is expressed in the subfractions of lipid droplets and the rest of the cytoplasm and AQP9 is constitutively expressed in the plasma membranes [2]. AQP3 and AQP7 facilitate glycerol outflow from adipocytes in response to β-adrenergic receptor-stimulated lipolysis (i.e., isoproterenol) via its translocation from the cytosolic fraction (AQP3) or lipid droplets (AQP7) to the plasma membrane [2,13,14]. Moreover, after acute leptin stimulation, AQP3 tends to surround lipid droplets more prominently, whereas AQP7 is translocated to the plasma membrane [13].

In fat depots, the expressions of these transmembrane channels show differences among anatomical locations. Internal fat depots present higher expression of AQP3 and AQP7 than subcutaneous depots do. This could reveal a greater lipolytic capacity in internal depots and a greater tendency to adipocyte hypertrophy in subcutaneous adipose tissue [13].

**Figure 1:**
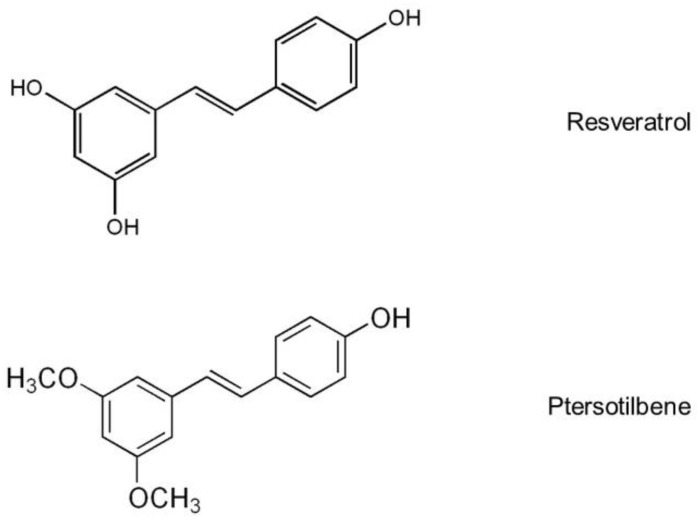
Resveratrol and pterostilbene chemical structure.

In recent years, great attention has been paid by the scientific community to phenolic compounds as potential active molecules useful for the prevention and treatment of obesity. A great number of studies has been carried out with resveratrol (3,5,4′-trihydroxy-*trans*-stilbene), a polyphenol belonging to the group of stilbenes, which shows three hydroxyl groups in its chemical structure (Figure 1). These studies have demonstrated that resveratrol, which shows anti-oxidant and anti-inflammatory activities, is able to prevent body fat accumulation induced by obesogenic diets in rodents. Several mechanisms of action, such as decreased adipogenesis, de novo lipogenesis and fatty acid uptake from circulating triglycerides, and increased lipolysis, fatty acid oxidation and thermogenesis have been revealed [15,16,17,18]. Most of these actions are mediated by the activation of SIRT1 and AMPK [17,18].

Resveratrol shows a low bioavailability due to its intense metabolism by phase II enzymes in the gut and liver [19]. Due to this fact, pterostilbene (4-[(E)-2-(3,5-dimethoxyphenyl) ethenyl]phenol) has been revealed as an interesting alternative. This is a dimethylether derivative of resveratrol, which shows a unique hydroxyl group (Figure 1). This structure confers to pterostilbene better biovailability and increased transport into cells [20,21]. Data concerning the effects of pterostilbene on obesity are scarce so far, but in a previous study carried out in our group we observed that it was able to reduce body fat accumulation in rats fed a high-fat high-sucrose diet, mainly by reducing de novo lipogenesis in white adipose tissue and increasing fatty oxidation in the liver [22,23]. Also, in genetically obese rats, we observed that pterostilbene increased the thermogenic and oxidative capacity of brown adipose tissue [24]. However, as far as we know, no data are available concerning the effects of resveratrol and pterostilbene on AQPs.

In this scenario, the aim of the present study was to determine whether the administration of resveratrol, and its methoxylated analogue pterostilbene, modifies AQPs expression in white and brown adipose tissues from rats fed an obesogenic diet, and to establish a potential relationship between these changes and the delipidating properties of these compounds.

## 2. Results

### 2.1. Adipose Tissue Weights

**Figure 2:**
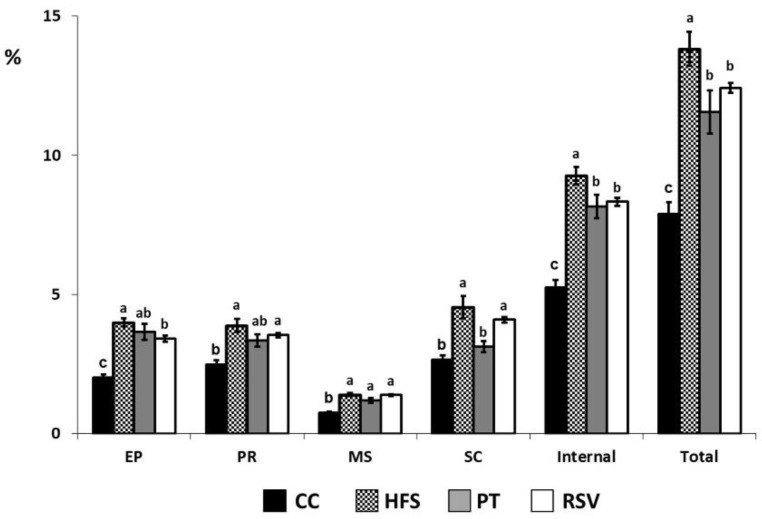
Adipose tissue weights, expressed as percentage of total body weight. Different letters indicate statistically significant differences. EP: epididymal adipose tissue, PR: perirenal adipose tissue, MS: mesenteric adipose tissue, SC: subcutaneous adipose tissue.

As expected, rats fed a high-fat, high-sucrose diet (HFS) showed higher adipose tissue weights relative to their body weight than those fed a standard diet (Control rats; CC) in all the depots analyzed. Pterostilbene (PT) prevented this increase in the case of subcutaneous adipose tissue and also when the weights of either the internal or all the dissected white adipose tissues were pooled. Resveratrol (RSV) prevented the increase in epididymal adipose tissue weight, as well as the weight of pooled internal depots or all the dissected white adipose tissues pooled. Nevertheless, the prevention in both PT and RSV groups was partial because a percent of adipose tissue weights in rats from these groups were higher than those in the control rats (Figure 2). No significant changes were found in interscapular brown adipose tissue (IBAT).

### 2.2. Serum Triglyceride Concentration

With regard to serum lipids, triglyceride values (mg/dL) were as follows: CC group: 47 ± 11; HFS: 60 ± 7; PT: 71 ± 10; RSV: 45 ± 2. Statistical differences were found only between both phenolic compounds.

### 2.3. Adipocyte Number and Size Determination

**Figure 3:**
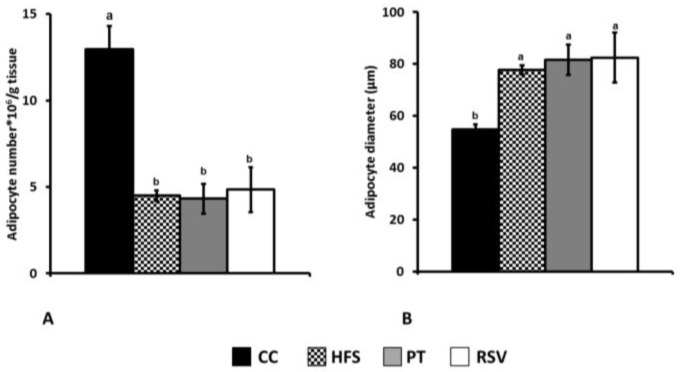
Adipocyte number (**A**) and size (**B**) in epididymal adipose tissue. Different letters indicate statistically significant differences.

**Figure 4:**
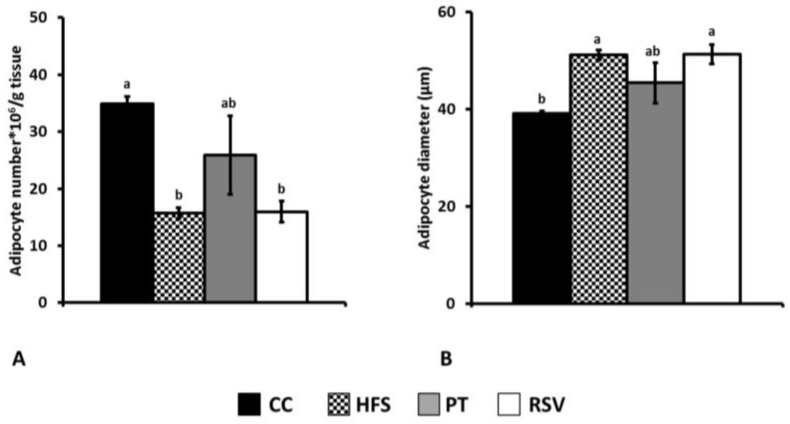
Adipocyte number (**A**) and size (**B**) in subcutaneous adipose tissue. Different letters indicate statistically significant differences.

In epididymal adipose tissue, CC group showed a bigger amount of adipocytes per gram of tissue having these cells a smaller diameter than in the other groups (Figure 3). In the subcutaneous depot, the results were similar to those observed in epididymal adipose tissue but, in this case, pterostilbene partially reversed the effects of the obesogenic diet feeding (Figure 4).

### 2.4. Gene and Protein AQP Expressions in Epididymal and Subcutaneous White Adipose Tissues

**Figure 5:**
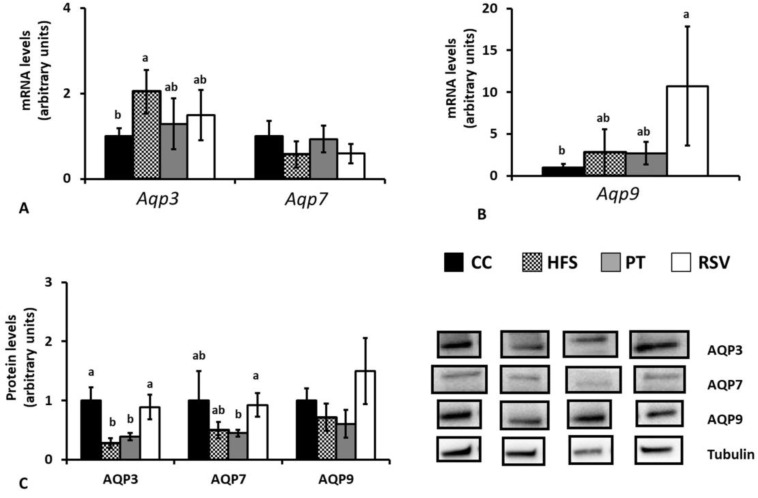
Epididymal adipose tissue aquaglyceroporin gene (**A**,**B**) and protein (**C**) expressions Different letters indicate statistically significant differences. *Aqp3* and AQP3: aquaglyceroporin 3, *Aqp7* and AQP7: aquaglyceroporin 7, *Aqp9* and AQP9: aquaglyceroporin 9.

In epididymal adipose tissue, *Aqp3* gene expression was significantly increased after HFS feeding (*p* < 0.05). Pterostilbene and resveratrol partially prevented this effect because rats treated with these compounds showed intermediate values between CC and HFS groups. No significant differences were found among experimental groups in *Aqp7* gene expression (Figure 5A). With regard to the *Aqp9* gene, neither HFS feeding, nor pterostilbene altered its expression. By contrast, resveratrol induced a great increase (Figure 5B).

When we measured protein expression of AQPs, AQP3 was significantly decreased by HFS feeding (*p* < 0.05), a situation that was totally reversed by resveratrol, but not by pterostilbene. No significant changes were induced by HFS feeding or phenolic compound administration in protein expressions of AQP7 and AQP9 (Figure 5C).

**Figure 6:**
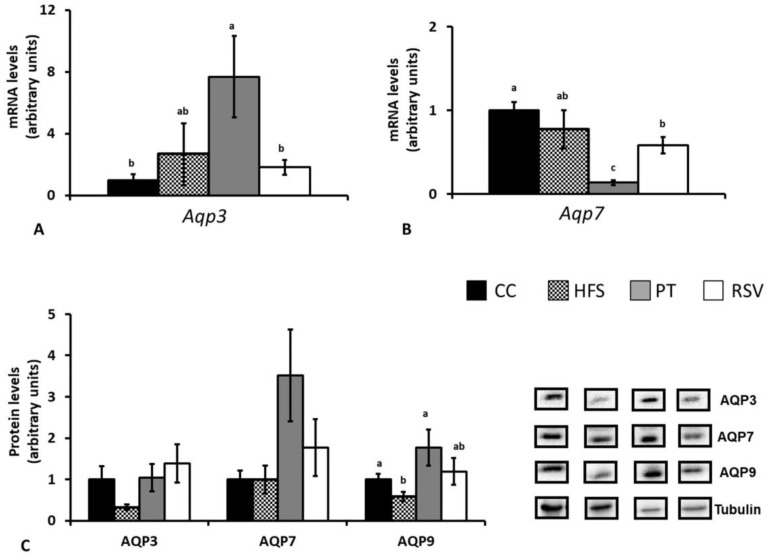
Subcutaneous adipose tissue aquaglyceroporin gene (**A**,**B**) and protein (**C**) expression. Different letters indicate statistically significant differences. *Aqp3* and AQP3: aquaglyceroporin 3, *Aqp7* and AQP7: aquaglyceroporin 7, *Aqp9* and AQP9: aquaglyceroporin 9.

The pattern of response of subcutaneous adipose tissue gene expression was different. In this tissue the only significant change found concerning *Aqp3* was the increase produced by pterostilbene (*p* < 0.01 vs. CC group) (Figure 6A). As far as *Aqp7* is concerned, the opposite effect was observed, which is to say that pterostilbene significantly decreased its gene expression (*p* < 0.001 vs. CC group) (Figure 6B). *Aqp9* gene expression was undetectable.

With regard to protein expression, no significant changes were observed among experimental groups in AQP3 and AQP7. By contrast, the reduction induced by HFS feeding in AQP9 was prevented by pterostilbene, but not by resveratrol (Figure 6C).

### 2.5. Gene and Protein AQP Expressions in Brown Adipose Tissue

**Figure 7:**
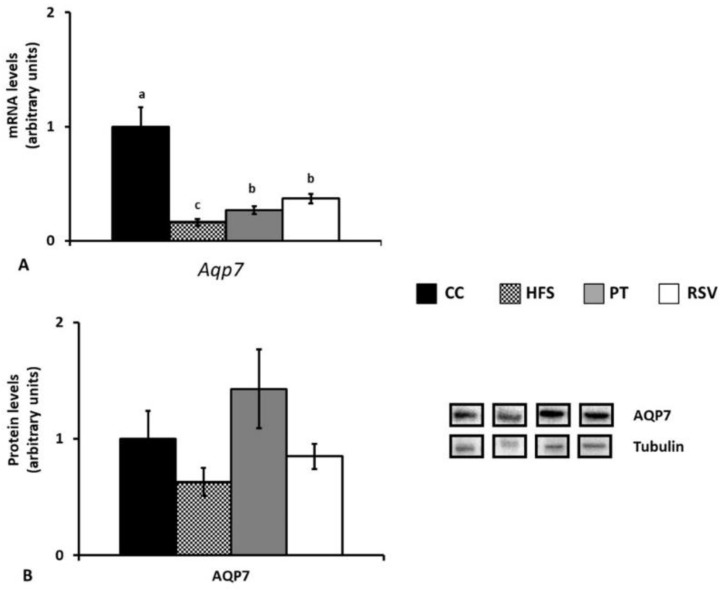
Interscapular brown adipose tissue gene (**A**) and protein (**B**) expressions. Different letters indicate statistically significant differences. *Aqp7* and AQP7: aquaglyceroporin 7.

In IBAT only AQP7 was detectable. HFS feeding significantly reduced gene expression (*p* < 0.001). This effect was partially prevented by both phenolic compounds (Figure 7A). The effects on gene expression were not translated into protein expression because no significant differences were observed among groups in this parameter (Figure 7B).

In order to better understand the effects of each dietary treatment a summary of all results concerning gene and protein AQP expressions is presented in Table 1. Finally, when Pearson r analysis was carried out, no significant correlations were observed either between adipocyte size and AQP gene expression or between adipocyte size and AQP protein expression.

## 3. Discussion

The prevalence of obesity and related co-morbidities, such as type 2 diabetes and NAFLD, has become a major health problem in Western societies. The discovery of AQPs in white adipose tissue has provided new perspectives about the mechanisms implicated in their etiopathogenia. Nevertheless, the effects of phenolic compounds on the modulation of AQPs has been studied very little so far. In fact, there is only one reported work that shows that the supplementation with apple polyphenols for 8 weeks induced increased AQP7 protein expression in adipocytes isolated from rat epididymal adipose tissue [25]. As far as we know, there are no reported data concerning the effects of resveratrol and pterostilbene on these proteins. In this context, we were interested in analyzing the effects of these phenolic compounds on AQPs (AQP3, AQP7 and AQP9) and their relationship with triglyceride accumulation in adipose tissues from rats fed an obesogenic diet.

Under our experimental conditions, as expected according to the literature [26,27,28,29], we observed that rats fed a high-fat high-sucrose diet for 6 weeks had larger white adipose tissues than rats fed a standard diet. This increase was due to hypertrophy, which significantly increased the size of adipocytes, as shown by their mean diameter, and this was observed in the experimental group. Consequently, the number of adipocytes per gram of tissue was reduced. The effects of both phenolic compounds on white adipose tissue were different. Thus, while pterostilbene partially prevented subcutaneous tissue accretion induced by the obesogenic diet, mainly by reducing adipocyte filling with triglycerides, resveratrol partially prevented epididymal tissue increase.

The main function of AQPs is the control of glycerol uptake and release, two key steps for triglyceride synthesis and hydrolysis, respectively [30]. Internal and subcutaneous white adipose tissues show different activities regarding these processes [31,32], as well as different expression of AQPs. Internal fat depots present a higher expression of AQP3 and AQP7, which are involved in glycerol release [13]. Therefore, in the present study we analyzed and compared the effects of dietary interventions on AQP3, AQP7, and AQP9 in subcutaneous and epididymal white adipose tissues, and also in IBAT adipose tissue.

In the epididymal depot this increase in tissue size, was accompanied by decreased AQP3 protein expression. When rats fed the obesogenic diet were treated with pterostilbene, a phenolic compound that did not avoid fat mass accumulation in this depot, the protein expression of this AQP remained unchanged. By contrast, when rats were treated with resveratrol, a polyphenol that partially avoided fat accumulation, AQP3 was increased. A similar pattern of response was followed by AQP7. These results suggest both that resveratrol may produce greater glycerol release from adipocytes, which is in good accordance with the increased lipolysis mediated by resveratrol reported in another study [16], and that this effect may contribute to the reduction in adipose tissue mass. The lack of change in AQP9 indicates that glycerol uptake seems not to be involved in the effects of dietary treatments on epididymal adipose tissue size.

A completely different situation took place in the subcutaneous adipose tissue. The increase in the size of this depot, induced by the obesogenic diet, was prevented by pterostilbene, but not by resveratrol. In this case, high-fat high-sucrose feeding did not lead to changes in AQPs involved in glycerol release (AQP3 and AQP7). By contrast, AQP9 was decreased, probably as a compensatory mechanism for further reducing glycerol uptake. As a logical result, in rats treated with pterostilbene, which were protected against diet-induced fat accumulation, this compensatory mechanism did not occur. However, in resveratrol-treated rats, which suffered an increase in this adipose depot similar to that found in HFS group, AQP9 remained elevated in order to partially counteract this fact. Consequently, it can be proposed that the reduction in glycerol uptake can form part of the mechanism underlying the fat-lowering effect induced by pterostilbene in subcutaneous adipose tissue. Taken as a whole, these results show a picture that is in good accordance with the fact that internal adipose tissues show greater lipolytic capacity, and thus greater expression of AQP3 and AQP7 [33]. In this line, it is important to remember that AQP9 is constitutively expressed in the plasma membrane of adipocytes and, contrary to AQP3 and AQP7, does not translocate to the cytoplasm and/or lipid droplets in response to lipolytic (isoproterenol) or lipogenic (insulin) stimuli [2].

Little is known so far about the importance of AQPs in brown adipose tissue metabolism. In the present study, as explained in the Results section, only AQP7 was detectable. The presence of AQP7 in BAT was firstly described by Skowronski and colleagues [34]. They found that capillary endothelium of BAT displayed prominent AQP7 staining, whereas AQP7 labelling in adipocyte membranes was undetectable. Due to the fact that no changes were observed among the four experimental groups in our study, unfortunately no clear conclusions can be obtained concerning the potential role of AQP7 in the analyzed dietary treatments in this tissue.

Throughout the manuscript we have observed that, as a general pattern, gene expression and protein expression do not respond in a similar way to the nutritional interventions. This phenomenon, previously observed by other authors [2,4], suggests the involvement of epigenetic mechanisms (i.e., DNA methylation, microRNAs) in the regulation of aquaglyceroporin translation. In fact, Boqué et al. [25] showed that high-fat high-sucrose feeding resulted in the methylation of *Aqp7* promoter region, whereas apple polyphenol administration reversed this effect. Also, and in line with these results, Agha et al. analyzed genome-wide DNA methylation profiles in the adipose tissue of 106 subjects, and they found that *Aqp7*methylation was strongly associated with excessive fat accumulation and visceral distribution [35]. Consequently, further epigenetic studies are needed to gain more insight concerning the regulation of AQPs by pterostilbene and resveratrol.

## 4. Material and Methods

### 4.1. Animals, Diets and Experimental Design

The experiment was conducted with thirty-six male Wistar rats with an initial body weight of 180 ± 2 g purchased from Harlan Ibérica (Barcelona, Spain), and in accordance with the University of the Basque Country’s Guide for the Care and Use of Laboratory Animals (Reference protocol approval CUEID CEBA/30/2010). The rats were individually housed in polycarbonate metabolic cages (Tecniplast Gazzada, Buguggiate, Italy) and placed in an air-conditioned room (22 ± 2 °C) with a 12 h light-dark cycle (lights off at 9:00 a.m.). After a 6-day adaptation period, the rats were randomly divided into 4 experimental groups of eight animals each. One group (control group; CC) was fed a commercial standard diet (TD.06416), which provided 3.7 kcal/g and 10% of calories as fat (Table 2) The other three groups, high-fat, high-sucrose group (HFS), resveratrol-treated group (RSV), and the pterostilbene-treated group (PT), were fed a commercial high-fat, high-sucrose diet (obesogenic diet; TD.06415) (Harlan Ibérica, Barcelona, Spain), which provided 4.6 kcal/g and 45% of kcal as fat (Table 2). In RSV and PT groups, resveratrol or pterostilbene were added to the fresh diet daily, in amounts that ensured a dose of 30 mg/kg body weight/day. Resveratrol was a generous gift from Monteloeder (Elche, Spain) and pterostilbene was purchased from Sigma (San Louis, MO, USA). All animals had free access to food and water. Food intake and body weight were measured daily. At the end of the experimental period (6 weeks) the animals were sacrificed, after a 12 h overnight fasting period, by cardiac exsanguination under anaesthesia (chloral hydrate). White adipose tissue from different regions (subcutaneous, epididymal, perirenal, and mesenteric) and interscapular brown adipose tissue were dissected and weighed, and then immediately frozen. All samples were stored at −80 °C until analysis.

### 4.2. Serum Triglyceride Concentration

Serum triglycerides were measured using a commercial kit (Spinreact, Barcelona, Spain).

### 4.3. DNA Extraction, Adipocyte Size, and Number Determination

Total DNA was isolated from epididymal and subcutaneous samples using the DNeasy Blood and Tissue Extraction Kit (Qiagen, Hilden, Germany), according to the manufacturer’s protocol. DNA quantification was carried out by NanoDrop 1000 (ThermoScientific, Waltham, MA, USA). The DNA was used for determination of diameter and number of adipocytes, assuming a lipid density of 0.915 (density of triolein), and a constant DNA content of 6.2 pg nucleus^−1^ [36].

### 4.4. Extraction and Analysis of RNA and Semi-Quantification by Reverse Transcription-Polymerase Chain Reaction (Real Time RT-PCR)

Total RNA was isolated from 100 mg of sample using Trizol (Invitrogen, Carlsbad, CA, USA), according to the manufacturer’s instructions. RNA samples were then treated with a DNA-free kit (Applied Biosystems, Austin, TX, USA) to remove any contamination with genomic DNA. The yield and quality of the RNA were assessed using a NanoDrop Spectrophotometer (Thermo Scientific, Wilmington, DE, USA). 1.5 µg of total RNA of each sample was reverse-transcribed to first-strand complementary DNA (cDNA) using iScript cDNA Synthesis Kit (Bio-Rad, Hercules, CA, USA).

*Aqp3*, *Aqp7*, and *Aqp9* were quantified, as was *18S*, which served as the reference gene. A 4.75 μL aliquot of each diluted complementary DNA sample was used for PCR amplification in a 12.5 μL reaction volume. The complementary DNA samples were amplified on a CFX96 real-time PCR detection system (Bio-Rad, Hercules, CA, USA) in the presence of SYBR^®^ Green master mix (Applied Biosystems, Foster City, CA, USA). Sequences of primers are given in Table 3 and the concentration of the primers was 300 nM. The PCR parameters were as follows: start at 50 °C for 2 min, denaturation at 95 °C for 10 min, denaturation at 95 °C for 15 s over 40 cycles (except 50 cycles for *Aqp7* in subcutaneous adipose tissue and 45 cycles for IBAT *Aqp7*), annealing temperatures are specified in Table 3, in all the cases the extension temperature was 60 °C. Gene expression analysis was performed using the comparative threshold cycle (Ct) method. Amplification of the *18SrRNA* sequence was performed in parallel and was used to normalize values obtained for target genes. The results were expressed as fold changes of the threshold cycle value relative to controls using the 2^−ΔΔCt^ method [37]. The specificity of a quantitative PCR assay was confirmed by dissociation curve.

### 4.5. Western Blot Analysis

For this purpose, 100 mg of tissue samples were homogenized in 500 µL of cellular PBS for white adipose tissue (pH 7.4), containing nuclease inhibitors, 100 mM phenylmethylsulfonyl fluoride and 100 mM iodoacetamide. The homogenates were centrifuged at 500× *g* for 10 min at 4 °C. Protein concentrations in homogenates were determined by using the Bradford method [38].

After protein quantification, immunoblot analyses were performed using 30 µg of protein from subcutaneous adipose tissue homogenate, 20 µg from epididymal adipose tissue homogenate and 40 µg from interscapular brown adipose tissue homogenate extracts, separated by electrophoresis in 10% SDS-polyacrylamide gel and transferred on to PVDF membranes. The membranes were then blocked with 5% casein PBS-Tween buffer for 1.5 h at room temperature. Subsequently, they were blotted with the following antibodies overnight at 4 °C: anti-aquaglyceroporin 3 (Abcam, Cambridge, UK), anti-aquaglyceroporin 7 (Santa Cruz Biotechnology, Dallas, TA, USA), anti-aquaglyceroporin 9 (Santa Cruz Biotechnology, Dallas, TA, USA). As secondary antibodies, we used anti-rabbit immunoglobulin G-horseradish peroxidase-linked (for AQP3, tubulin) and anti-mouse immunoglobulin G-horseradish peroxidase-linked (for AQP7 and AQP9) antibodies. Densities were analyzed by a ChemiDoc MP imaging system (BioRad, Hercules, CA, USA). For densitometric quantification, levels of each protein for each sample were normalized to loading controls (tubulin (Cell Signaling Technology, Danvers, MA, USA)).

### 4.6. Statistical Analysis

The results are presented as means ± standard error of the means. Statistical analysis was performed using SPSS 23.0 (SPSS Inc., Chicago, IL, USA). All the parameters were normally distributed according to the Shapiro–Wilks test. Then, data were analyzed by using one-way ANOVA followed by the Newman–Keuls post-hoc test. Pearson’s r correlations were used to assess the potential relationships and associations between adipocyte size and aquaglyceroporins gene and protein expressions. Statistical significance was set-up at *p* < 0.05.

## 5. Conclusions

In summary, under overfeeding conditions, pterostilbene reduces subcutaneous adipose tissue weight and prevents the decrease in AQP9 induced by obesogenic feeding, and thus the uptake of glycerol for triglyceride accumulation. Resveratrol reduces epididymal adipose tissue weight and avoids the decrease in AQPs related to glycerol release induced by high-fat high-sucrose feeding, suggesting the involvement of lipolysis in its obesity prevention effect.

## Figures and Tables

**Table 1:** Summary of the changes in gene and protein expressions of the analyzed aquaglyceroporins in epididymal and subcutaneous white adipose tissues and interscapular brown adipose tissue.

Adipose Tissue Depot	mRNA Expression	Protein Expression
**Epididymal Adipose Tissue**		
AQP9	CC: NS	CC: NS
	PT: NS	PT: NS
	RSV: NS	RSV: NS
AQP7	CC: NS	CC: NS
	PT: NS	PT: NS
	RSV: NS	RSV: NS
AQP3	CC: ↑ 50%	CC: ↑ 100%
	PT: NS	PT: NS
	RSV: ↑ 50%	RSV: ↑ 100%
**Subcutaneous Adipose Tissue**		
AQP9		CC: ↑ 70%
	Not detected	PT: ↑ 200%
		RSV: NS
AQP7	CC: NS	CC: NS
	PT: ↓ 80%	PT: NS
	RSV: NS	RSV: NS
AQP3	CC: NS	CC: NS
	PT: NS	PT: NS
	RSV: NS	RSV: NS
**Interscapular Brown Adipose Tissue**		
AQP9		
	Not detected	Not detected
	
AQP7	CC: ↑ 80%	
	PT: ↑ 70%	NS
	RSV: ↑ 100%	
AQP3		
	Not detected	Not detected
	

Changes in HFS group are expressed versus the control group and changes in PT and RSV groups are expressed versus HFS group. AQP: aquaglyceroporins; HFS: high-fat, high-sucrose group; NS: no significant; PT: pterostilbene group; RSV: resveratrol group; ↑ increase; ↓ reduction.

**Table 2:** Composition of experimental diets. Values represent the percentages of each nutrient per total kilocalories.

Macronutrient	Standard Diet	Obesogenic Diet
Protein	20%	19%
Carbohydrates	70%	36%
Fat	10%	45%
	29% saturated	36% saturated
	37% monounsaturated	47% monounsaturated
	34% polyunsaturated	17% polyunsaturated

**Table 3:** Primer sequences and annealing temperatures for RT-PCR amplification of each gene studied.

Gene	Sense Primer Sequence	Antisense Primer Sequence	Annealing Temperature	Genbank Number
*Aquaporin 3*	5′-CCCCTTGTGATGCCTCTC-3′	5′-CCCTAGCTGGCAGAGTTC-3′	EP 57.8 °C, SC 60 °C	NM_031703.1
*Aquaporin 7*	5′-ATCCTTGTTTGCGTTCTTGG-3′	5′-GCGTGAATTAAGCCCAGGTA-3′	EP 60 °C, SC 65.6 °C, IBAT 67 °C	NM_019157.2
*Aquaporin 9*	5′-CTCAGTCCCAGGCTCTTCAC-3′	5′-ATGGCTCTGCCTTCATGTCT-3′	EP 60 °C	NM_022960.2
*18Sr RNA*	5′-GTGGGCCTGCGGCTTAAT-3′	5′-GCCAGAGTCTCGTTCGTTATC-3′	60 °C	M11188.1

*18Sr*: 18S ribosomal, SC: subcutaneous adipose tissue, EP: epididymal adipose tissue, IBAT: interscapular brown adipose tissue.
